# Supplementary material for: Factors associated with mortality of elderly people due to COVID-19: Protocol for systematic review and meta-analysis
Source: PLoS One. 2024 Apr 18;19(4):e0289576. doi: 10.1371/journal.pone.0289576 (PMC11025961; doi:10.1371/journal.pone.0289576)
Supplement: S1 Table — (DOCX) [file pone.0289576.s001.docx]

**S1 Table. List of abbreviations used in the text.**

| Abreviaturas | |
| --- | --- |
| PROSPERO | International Prospective Register of Systematic Reviews |
| PRISMA | Preferred Reporting Items for Systematic Reviews and Meta-Analyses Protocols |
| PICOS | Population, exposure, outcome, and types of studies |
| MeSH | Medical Subject Headings |
| UFRN | Federal University of Rio Grande do Norte |
| QCRI | Qatar Computing Research Institute |
| GRADE | Grading of recommendations, assessment, development and evaluation |
| NHLBI | National Heart, Lung, and Blood Institute |
| RevMan | ReviewManager |
