# Supplementary material for: Factors associated with mortality of elderly people due to COVID-19: Protocol for systematic review and meta-analysis
Source: PLoS One. 2024 Apr 18;19(4):e0289576. doi: 10.1371/journal.pone.0289576 (PMC11025961; doi:10.1371/journal.pone.0289576)
Supplement: S1 File — From: Page MJ, McKenzie JE, Bossuyt PM, Boutron I, Hoffmann TC, Mulrow CD, et al. The PRISMA 2020 statement: an updated guideline for reporting systematic reviews. BMJ 2021;372: n71. doi: 10.1136/bmj.n71. (DOCX) [file pone.0289576.s002.docx]

**S1 File.** **PRISMA study selection flowchart.**

Records removed *before screening*:

Duplicate records removed (n = )

Records marked as ineligible by automation tools (n = )

Records removed for other reasons (n = )

Records identified from*:

Databases (n = )

Registers (n = )

**Identification**

Records screened

(n = )

Records excluded**

(n = )

Reports sought for retrieval

(n = )

Reports not retrieved

(n = )

**Screening**

Reports assessed for eligibility

(n = )

Reports excluded:

Reason 1 (n = )

Reason 2 (n = )

Reason 3 (n = )

etc.

Studies included in the review

(n = )

Reports of included studies

(n = )

**Included**

*From:*  Page MJ, McKenzie JE, Bossuyt PM, Boutron I, Hoffmann TC, Mulrow CD, et al. The PRISMA 2020 statement: an updated guideline for reporting systematic reviews. BMJ 2021;372: n71. doi: 10.1136/bmj.n71
