# Supplementary material for: Factors associated with mortality of elderly people due to COVID-19: Protocol for systematic review and meta-analysis
Source: PLoS One. 2024 Apr 18;19(4):e0289576. doi: 10.1371/journal.pone.0289576 (PMC11025961; doi:10.1371/journal.pone.0289576)
Supplement: S3 File — Own authorship. (DOCX) [file pone.0289576.s004.docx]

**S3 File. Search equation to be used in each database.**

| **Search equation** |
| --- |
| mortality AND (covid-19 OR sars-cov-2) AND aged AND “risk factors” |

Own authorship.
