# Supplementary material for: Factors associated with mortality of elderly people due to COVID-19: Protocol for systematic review and meta-analysis
Source: PLoS One. 2024 Apr 18;19(4):e0289576. doi: 10.1371/journal.pone.0289576 (PMC11025961; doi:10.1371/journal.pone.0289576)
Supplement: Attachment — Submitted filename: Response to Reviewers.docx [file pone.0289576.s005.docx]

**Response to Reviewers**

*Dear reviewers, below we send you our thoughts on your comments. Thank you in advance for your cooperation.*

**2. Is the protocol technically sound and planned in a manner that will lead to a meaningful outcome and allow testing the stated hypotheses?**

The manuscript should describe the methods in sufficient detail to prevent undisclosed flexibility in the experimental procedure or analysis pipeline, including sufficient outcome-neutral conditions (e.g. necessary controls, absence of floor or ceiling effects) to test the proposed hypotheses and a statistical power analysis where applicable. As there may be aspects of the methodology and analysis which can only be refined once the work is undertaken, authors should outline potential assumptions and explicitly describe what aspects of the proposed analyses, if any, are exploratory.

Reviewer #1: Yes

Reviewer #2: Yes

Reviewer #3: Partly

*Thank you for taking the time to review our manuscript and for your comments. Considering the relevance of summarizing the current global evidence on biological and non-biological factors associated with the mortality rate of the elderly due to Covid-19, we conducted our research questions according to the dimensions explored in the construction of the review. The research questions, in addition to being included in the registered PRISMA-P protocol, are fully described in the methodology of the manuscript, and were constructed in accordance with the justification presented in the Introduction section.*

**5. Is the manuscript presented in an intelligible fashion and written in standard English?**

PLOS ONE does not copyedit accepted manuscripts, so the language in submitted articles must be clear, correct, and unambiguous. Any typographical or grammatical errors should be corrected at revision, so please note any specific errors here.

Reviewer #1: Yes

Reviewer #2: Yes

Reviewer #3: No

*We would like to inform you that we have made some adjustments to improve the fluidity and clarity of the English text.*

**6. Review Comments to the Author**

**Reviewer #1: Dears,**

**The paper has well-designed research methods, appropriate statistical analysis and a relatively good interpretation of the results.**

**-Please be sure to use only keywords accordingly to medical subject headings (Mesh word) for a better indexing.**

**I suggest you add a table with the list of abbreviations used in the text.**

**I suggest you implement the abstract in order to make it more understandable to authors.**

**The introduction should be expanded perhaps by adding a section on temporomandibular disorders. I recommend some references:[10.3390/jcm12072652];[10.1111/joor.13496]**

**The conclusion is in accordance with the objectives of the research, its results and their interpretation, as well as the relevant literature.**

*All the terms entered in the search key have been reviewed and are in MESH as descriptors or between terms.*

*The table with the list of abbreviations was inserted in the article.*

*We understand that temporomandibular disorders are not part of the scope of this review, as an outcome or criterion to be investigated. Therefore, we do not consider it necessary to include a section on the subject in the introduction.*

**Reviewer #2: The present work is very thorough and detailed.**

**I would like to ask the author why they removed the hospitalized elderly in the exclusion criteria, considering that most covid deaths occurred in hospital facilities. Modify the conclusions to strengthen the sense of the review.**

*In agreement with the reviewer's analysis, the group also understands that hospitalized elderly people should not be considered as exclusion criteria. Criteria modified in the text.*

**Reviewer #3: The authors present a systematic review protocol with the objective of evaluating factors associated with higher mortality from Covid-19 in the elderly.**

**Although the authors state in the introduction "there is still no systematic and comprehensive evidence worldwide on such issues specifically with elderly people" (lines 113 and 114), a quick search finds systematic reviews with meta-analysis with the same objective already published (doi: 10.1177/23337214211057392./doi: 10.3390/ijerph18158008/ https://doi.org/10.1556/2060.2022.00206) and the authors need to point out in the protocol whether what they propose may indicate different results from those published.**

**Finally, the protocol needs to consider the possibility of meta-regression in the methodology as it is a systematic review of observational studies, may have a shorter and more objective introduction and needs to be reviewed by a native English speaker to correct grammatical errors. As a result, there is no quality or originality for the protocol to be published.**

*The studies pointed out by the reviewer are relevant publications in the field and appear to be related to the topic agreed in this protocol.*

*However, our review aims to cover some of the methodological limitations presented in the articles cited and other studies carried out, such as: limitation of research time, restricted number of databases, restriction to the investigation of local data (one or a few countries) and research limited to biological aspects.*

*In this sense, we believe that the review proposed in our protocol is unprecedented in that it expands the investigation of mortality to include clinical (biological) and behavioral (non-biological) aspects, without restricting the location or language, with an extended time frame (2020-2023), considering classic epidemiological designs and with searches in an expanded number of databases.*

*Metaregression was considered in the topic of data analysis.*

*The article was revised again to adjust grammatical errors.*

*Respectfully,*

*The authors.*
