# Supplementary material for: Factors associated with mortality of elderly people due to COVID-19: Protocol for systematic review and meta-analysis
Source: PLoS One. 2024 Apr 18;19(4):e0289576. doi: 10.1371/journal.pone.0289576 (PMC11025961; doi:10.1371/journal.pone.0289576)
Supplement: Attachment — Submitted filename: Response to Reviewers.docx [file pone.0289576.s006.docx]

**Federal University of Rio Grande do Norte**

**Post-Graduation Program in Public Health**

Av. Senador Salgado Filho, 1787 – Lagoa Nova

ZIP: 59056-000 – Natal, RN, Brazil

Feb 23th, 2024

Rebuttal Letter for PLOS ONE

Thank you for considering our manuscript for publication at PLOS ONE.We are confident in PLOS ONE's reputation and dedication to scientific excellence, and hope that the revisions we have made to our manuscript are in line with the required publication standards. We believe that the suggestions contribute significantly to the quality and clarity of our article.

The responses to each of the suggestions are detailed at the end of this message.

Once again, we thank you for your consideration of our work and we are at your disposal for any further clarification that may be required.

Sincerely,

**Reviewer #2: Dears colleagues**

**The paper has well-designed research methods, appropriate statistical analysis and a relatively good interpretation of the results.**

**Please be sure to use only keywords accordingly to medical subject headings (Mesh word) for a better indexing.**

**I suggest you implement the abstract in order to make it more understandable to authors.**

**The introduction should be expanded perhaps by adding a section on covid diseases.**

**The conclusion is in accordance with the objectives of the research, its results and their interpretation, as well as the relevant literature.**

*All the terms entered in the search key have been reviewed and they are MESH as descriptors or between terms.*

*The summary has been improved.*

*As suggested, a paragraph has been added to the introduction about the COVID-19 disease.*

**Reviewer #3: The authors responded satisfactorily to the reviewers' recommendations and managed to improve the justification for yet another protocol on the topic in the introduction, including what it adds to what has already been published in other reviews. However, there are still corrections to be made.**

**In the introduction, after the phrase "Despite the wealth of studies produced and published", authors must cite the studies (doi: 10.1177/23337214211057392./doi:10.3390/ijerph18158008/ https://doi.org/10.1556/2060.2022.00206).**

**In the methodology, the text on meta-regression was inserted in Portuguese "Além disso, uma metaregressão poderá complementar a exploração das fontes da heterogeneidade.", and the main reason for considering metaregression in this protocol is not to explore sources of heterogeneity, but rather to allow the analysis of confounding factors associated with mortality in the elderly, allowing us to identify which are truly associated with COVID-19. They must correct and improve the text in English!**

*We have included the citation to the studies after the sentence, as suggested.*

*We have revised the meta-regression sentence and we enhanced the English text.*

**Reviewer #4: Comments to authors**

**Thank you for the opportunity to review (R1) of the manuscript titled “Factors associated with mortality of elderly people due to COVID-19: Protocol for systematic review and meta-analysis.”**

**Overall, the findings of this worldwide comprehensive study will benefit in planning and making targeted interventions on elderly people to reduce mortality due to COVID-19. Moreover countries will minimize the influences of pandemics / health crises on elder population.**

**I believe there are chances to more strengthen this manuscript. Here are the comments to be addressed by the authors.**

**1. Title**

**-Please write the word covid-19 in capital letters, and assure that it is consistent through the manuscript**

**2. Abstract**

**- Well written.**

**-Please specify the version of the software will be used in this meta analysis?**

**3. Introduction**

**- Well written.**

**4. Methods and Methods**

**-In the exclusion criteria what is the reason in which the ahuthors will exclude Cross-sectional observational studies? Remember that currently cross sectional studies that can assess the prevalence and associated factors of mortality among elderly people due to COVID-19 are available. Please justify?**

**- In the data synthesis and analysis section, the stetment “ A quantitative synthesis of the results will be performed if the included studies are sufficiently homogeneous (75 to 100%) is not clear.**

**Please clarify?**

**-What is the statement “Além disso, uma metaregressão poderá complementar a exploração das fontes da heterogeneidade”? If it is meaningful please write in English or remove it.**

**-Please specify the methods of assessing the source of heterogeneity in this meta analysis. Subgroup analyses or meta regression?**

**- The author should specify how publcation bias will be assessed?**

**-Also the author should propose to see the small study effects or sensitivity analysis?**

**5. Discussion**

**- Well written.**

**6. Conclusion**

**- Well written.**

**7. Bibliographic references**

**- Well written.**

*We have capitalized the term COVID-19 consistently throughout the manuscript, as per the suggestion*.

*With regard to the criterion for excluding cross-sectional studies, we believe that these studies collect data at a single point in time and therefore have limitations in terms of causal inference and temporal follow-up. Consequently, we chose to take advantage of the benefits offered by longitudinal studies in this context. This becomes particularly advantageous when considering that certain COVID-19 patients have undergone significant follow-up, which may facilitate the identification of additional factors associated with mortality from the disease. In addition, we postulate that longitudinal studies are able to provide associated factors, aligning with the objectives of our protocol. Specifically, cohort studies not only provide associated factors, but also delve into risk factors. Given the possibility of data homogeneity, this increases our ability to carry out a more robust meta-analysis.*

*Regarding the quantitative synthesis section, we would like to inform you that a meta-analysis will be carried out if the studies show sufficient homogeneity. We have revised the sentence in the manuscript.*

*We have revised the meta-regression sentence and enhanced the English text.*

*The funnel plot and/or sensitivity analysis will be used to assess publication bias. Added to the study, according to the reviewer's observation.*

**Reviewer #5: The article is well written. The authors have taken the reviewers' comments into account. My additions to the article are:**

**Introduction**

**End the introduction with a sentence specifying the objective of the systematic review.**

**Methods**

**1. Evaluating patient mortality covid-19, in data extraction, sars- cov 2 variants also seems relevant during extraction**

**2. In the data analysis section, there is a sentence in Portuguese that needs to be translated into English “Além disso, uma metaregressão poderá complementar a exploração das fontes da heterogeneidade “ .**

**3. Specify the level of heterogeneity (low, moderate and high) with the I2 values and what you will do if the heterogeneity is high, e.g. sensitivity or subgroup analysis or metaregression.**

**4. Add a section on publication bias, e.g. Egger's test or Begg's funnel plot.**

**5. Add a sentence on ethics statement, although ethics committee approval is not required for meta-analysis.**

**Discussion**

**Add strenghs and limitations of the sudy, Implications of the results you find**

*We have added the sentence with the objective of the review to the end of the introduction.*

*We have added the types of variants studied to the data extraction.*

*We have revised the meta-regression sentence and enhanced the English text.*

*We have added to the text the values to be considered in the heterogeneity with I²: near 25% will be considered low, near 50%, moderate, and near 75%, high.*

*We have revised the meta-regression sentence and enhanced the English text.*

*The funnel plot and/or sensitivity analysis will be used to assess publication bias. Added to the study, according to the reviewer's observation.*

*A section on ethics statements was included.*

*We have added a section on the strengths, limitations and implications of the study at the end of the discussion, as suggested.*

*Respectfully,*

*The authors.*
